# Wild Cane Toads (*Rhinella marina*) Expel Foreign Matter from the Coelom via the Urinary Bladder in Response to Internal Injury, Endoparasites and Disease

**DOI:** 10.1371/journal.pone.0134036

**Published:** 2015-08-12

**Authors:** Crystal Kelehear, Hugh I. Jones, Benjamin A. Wood, Richard Shine

**Affiliations:** 1 School of Biological Sciences, The University of Sydney, Camperdown, NSW, Australia; 2 School of Pathology and Laboratory Medicine, The University of Western Australia, Crawley, WA, Australia; 3 PathWest Laboratory Medicine, Queen Elizabeth Medical Centre, Nedlands, WA, Australia; Bigelow Laboratory for Ocean Sciences, UNITED STATES

## Abstract

Dissections of >1,200 wild-caught cane toads (*Rhinella marina*) in tropical Australia confirm a laboratory report that anurans can expel foreign objects from the coelom by incorporating them into the urinary bladder. The foreign objects that we found inside bladders included a diverse array of items (e.g., grass seeds, twigs, insect prey, parasites), many of which may have entered the coelom via rupture of the gut wall. In some cases, the urinary bladder was fused to other organs including liver, fat bodies, ovaries, Bidder’s organs, lungs, mesentery, stomach wall, gall bladder, and the abdominal wall. Acanthocephalan parasites (of a range of developmental stages) were identified from the walls of the urinary bladders of three cane toads. This organ may play a significant role in destroying or excreting metazoan parasites, as well as inanimate objects.

## Introduction

A recent experimental study documented a remarkable mechanism by which anuran amphibians can expel foreign objects from their coelom: the objects (surgically implanted beads and radiotelemetry transmitters) can be incorporated into the bladder, and later expelled with urine [1]. This route of object expulsion was reported in Australian native frogs (*Litoria* spp. and *Cyclorana australis*) and in invasive cane toads (*Rhinella marina*, previously *Bufo marinus*; [1]). This pathway of expulsion may well be adaptive [1] but its biological relevance (and frequency under natural conditions) remains unclear; plausibly, however, an anuran could benefit from such an ability. Anuran amphibians run a high risk of traumatic injury. For example, at the completion of a frog’s leap, the ventral surface contacts the ground with considerable force [2,3], raising the dangerous prospect of the skin being penetrated by sharp objects. Further, amphibians swallow their prey whole and often still alive; so the sharp chitinous body parts of an ingested insect may pierce the gastrointestinal tract and enter the coelom. In addition, amphibians are exposed to many parasites that burrow through viscera to reach their infection sites. For example, infective larvae of the lung parasite (*Rhabdias pseudosphaerocephala*) can enter their toad host through the eye socket and burrow through the subcutaneous tissue, musculature, and coelom *en route* to the lungs, leaving a trail of inflammation [4]. Because the urinary bladder of anurans is very large and thin-walled, foreign objects in the coelom are more likely to contact the bladder than any other organ.

The first step in clarifying the biological role of this mechanism is to identify the kinds of foreign objects that are incorporated and expelled in this way. While dissecting cane toads in the Australian tropics for parasitological research (e.g., [5,6]), we observed gross abnormalities in the urinary bladders of several animals. Further, during the course of a laboratory-based immunological study (Kelehear *et al*. in prep.) we noted that experimentally-injected toads exhibited higher rates of bladder abnormalities than did field-caught animals. Although our laboratory study was not specifically designed to investigate the role of the toad bladder in expulsion of foreign objects (and hence, for example, we did not design our injection experiments to elucidate the mechanisms involved), our data nonetheless provide the most extensive information yet available on this topic. In the present study, we examined the incidence and form of bladder abnormalities to determine their cause, and hence test the hypothesis that the urinary bladder plays a functional role in the destruction or excretion of internal parasites and other foreign matter.

## Materials and Methods

### Ethics statement

This study was carried out in strict accordance with the recommendations in the Australian Code of Practice for Care and Use of Animals for Scientific Purposes of the National Health and Medical Research Council. All procedures were approved by the University of Sydney Animal Ethics Committee (L04/4-2008/2/4788; L04/5-2010/2/5334; L04/1-2010/3/5193). Land owners, or their representatives, provided permission for access to private lands. The study species is an invasive species and is not listed as endangered or protected in Australia.

### Field dissections

A total of 1,254 cane toads were collected from the wild over the period 10 September 2008–11 June 2011 at five sites (12.4127°S, 130.8578°E; 12.4878°S, 130.9669°E; 12.6218°S, 131.3047°E; 12.6510°S, 131.3185°E; 12.714°S, 131.4197°E) distributed between Darwin and Leaning Tree Lagoon, Northern Territory, Australia. All toads were humanely euthanized using an overdose of sodium pentobarbital, dissected, and inspected for internal traumatic injuries and gross abnormalities involving the urinary bladder (*e*.*g*., adhesions, inclusions, cysts). For seven toads with these latter abnormalities, we preserved the affected organs in 10% neutral buffered formalin for histology. Serial sections were cut at 6 μm, and stained with hematoxylin and eosin for histological examination.

### Laboratory manipulations

In addition, 20 toads were collected from each of two of the above sites (12.48783°S, 130.9669°E and 12.4127°S, 130.8578°E) over the period 17–22 February 2011 and maintained in the laboratory for 41 days. Over this period, toads were subjected to 3–5 cardiac punctures to obtain 0.75 mL of blood and one intracoelomic injection with sheep red blood cells (SRBC; *n* = 40; as a component of another study: Kelehear *et al*. in prep.). At the completion of the experiment all toads were humanely euthanized using an overdose of sodium pentobarbital, dissected and examined macroscopically for bladder abnormalities. All affected organs were excised and prepared for histological examination as above.

## Results

### Traumatic internal injuries

Of the field-collected toads, only four (0.3%) had overt traumatic internal injuries. One very large (477 g) female toad had a large puncture wound (approx. 10 mm diameter) piercing her side clean through to her large intestine, leaving her gastrointestinal tract and coelom open to the environment. The wound was highly malodorous and her liver, right fat body, and wall of the large intestine were fused to the abdominal wall at the puncture site. One male toad had a hole (of unexplained origin) in his stomach wall leading through to the coelom. Another toad contained a wasp (approx. 1.5 cm long) that had escaped the stomach and embedded its mouthparts in the toad’s right lung. There was a hole in the stomach wall and the adjacent lung through which the wasp was positioned, with its posterior still inside the stomach and its head inside the lung. Another toad had large sharp grass seeds in the coelom; some of these seeds had pierced the right lung (which was deflated with no elasticity) and were partially engulfed by the urinary bladder; additional seeds were free inside the bladder.

### Bladder abnormalities

**Fig 1:**
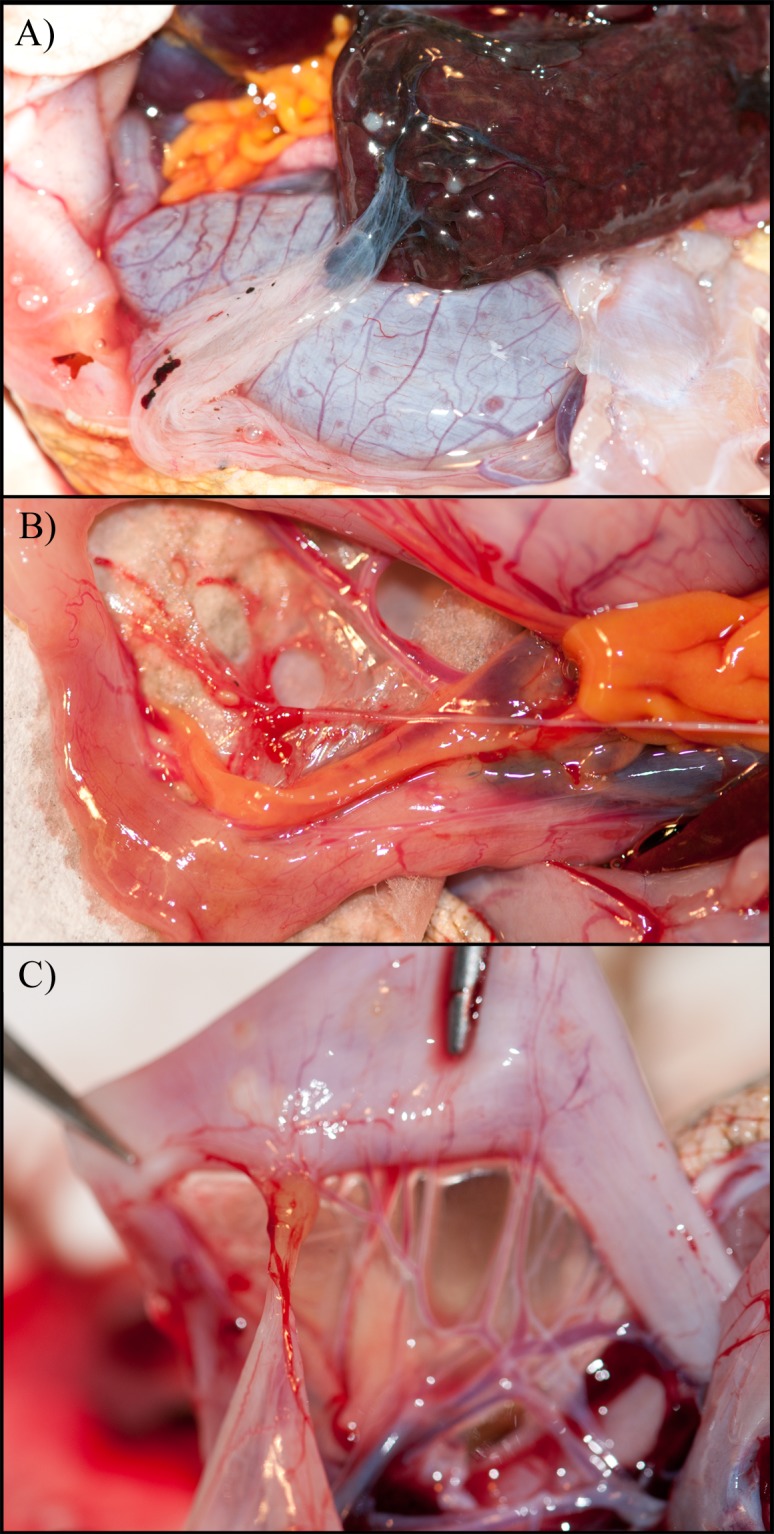
Gross appearance of abnormalities involving fusion of the cane toad urinary bladder with other organs. A) Urinary bladder fused to liver, note adjacent cysts on liver tissue and dark intrusion inside the lumen of the urinary bladder. B) Urinary bladder fused to mesentery via thin string, note adjacent cysts on mesentery. C) Urinary bladder fused to Spirurid cyst on stomach wall.

Eighteen (1.4%) of the field-collected cane toads possessed macroscopically evident fusions of the urinary bladder to other organs. These abnormalities were more common in the experimental toads, with seven (17.5%) of the toads subjected to intracoelomic needle penetration possessing bladder abnormalities at dissection (S1 Table). Eighteen (1.3%) of all dissected toads had cysts on the wall of the urinary bladder. Fusions between the urinary bladder and other organs involved a range of organs including liver, fat bodies, ovaries, Bidder’s organs, lungs, mesentery, stomach wall, gall bladder, and the abdominal wall (S1 Table, Fig 1). In eight of these cases the involved organs exhibited growths and/or discoloration (S1 Table). In eight toads the urinary bladder was fused either directly or immediately adjacent to parasitic cysts or live parasites (S1 Table, Fig 1). In one case, the area of fusion showed fat necrosis and xanthogranulomatous cystitis with hemosiderin deposition and lymphoid aggregates in the urinary bladder wall (S1 Table). The fusions sometimes involved a thin string (fine fibrous tract) of the urinary bladder attached to another organ; in one case this string contained hemosiderin-laden macrophages, and in another, there were focal lymphoid aggregates (S1 Table). Several other bladders contained lymphoid aggregates, but no foreign material was visible. In another fusion between the urinary bladder and the liver, there was prominent hemosiderin deposition with numerous macrophages.

The lumen of one bladder contained grass seeds (above), others contained blades of grass and small twigs, and one contained a hard and calcified clump of leaves and sticks. The lumens of several bladders contained amorphous masses and lumps of fat.

**Fig 2:**
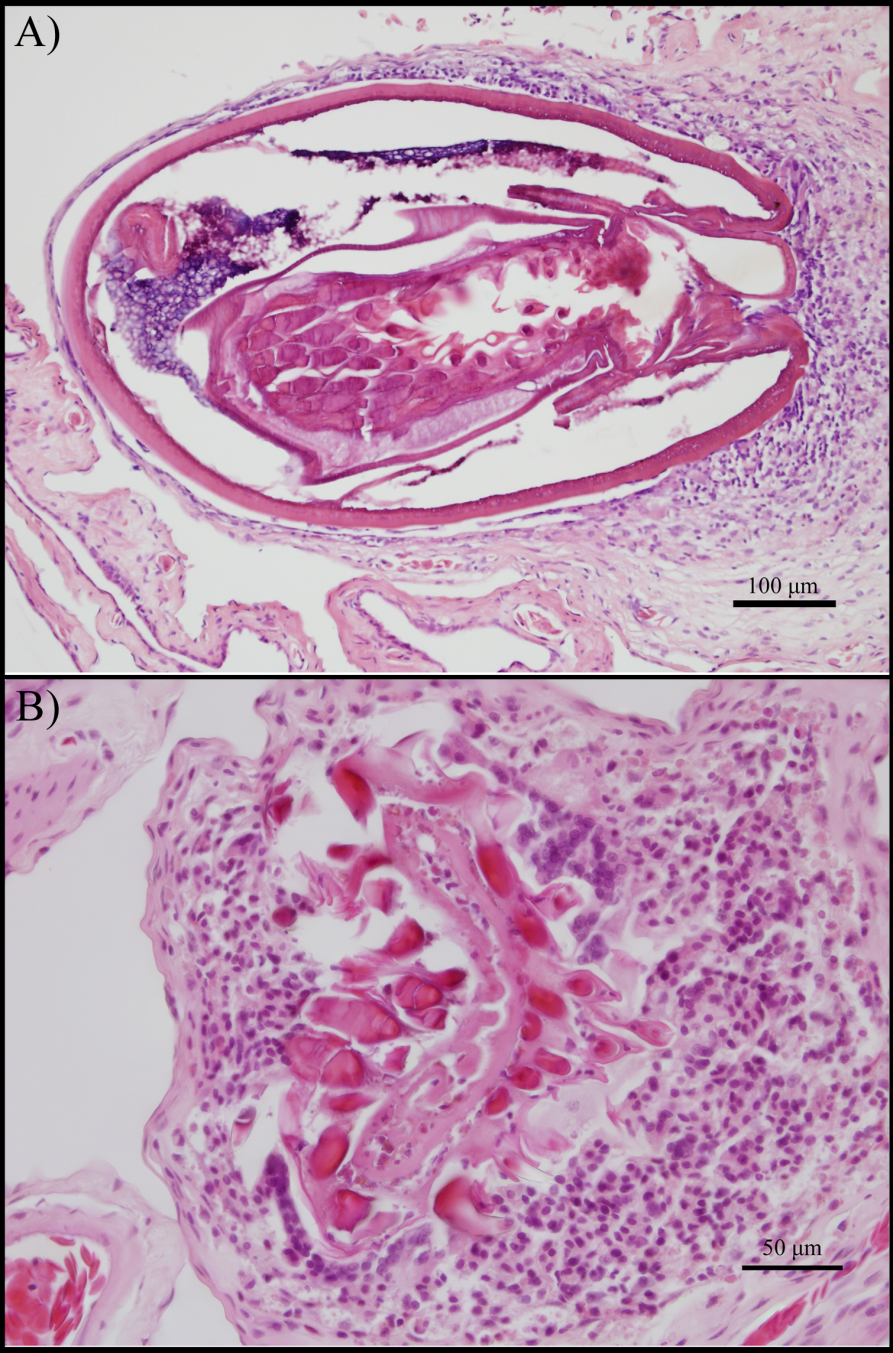
Histology of the cane toad urinary bladder walls containing acanthocephalans. A) Cystacanth larva within the bladder wall, note spines inverted. B) Everted acanthocephalan spines embedded in inflammatory cells. Both images are focus-stacked from three photographs to maximize depth of field.

Of the 18 toads that possessed cysts on the bladder walls, we inspected six histologically. Microscopic examination showed larval acanthocephalans in three of these. One bladder wall contained an Acanthocephalan cystacanth (Fig 2A). The wall of the cyst was hyaline, with the spiny proboscis invaginated, with a moderate lymphohistiocytic inflammatory reaction present at the anterior pole. The same bladder contained several acanthocephalan spines that were everted and embedded in a mass of inflammatory cells (Fig 2B). In addition, there were two cysts, with concentric layers of inflammatory cells and fibroblasts, containing the trunk of larval Acanthocephala. These were in the process of being resorbed with the structures of internal organs outlined by degenerate cells. A second toad bladder contained everted acanthocephalan spines in the process of being resorbed, also surrounded by a thick layer of concentric inflammatory cells. A third bladder wall contained an oblique section of the trunk of an acanthocephalan, again with the degenerate cells outlining the organs (S1 Table).

## Discussion

The capacity to eliminate foreign matter from the abdominal cavity has been described in several species of ectotherms, including amphibians [1], reptiles [7,8] and fish [9,10,11,12,13]. The specific structures used to expel foreign objects differ among these groups; for example, snakes utilize the gut whereas anurans utilize the bladder. Our field data confirm a previous laboratory report that the urinary bladder of cane toads is able to engulf foreign matter from the coelom, presumably to be expelled to the environment with urine [1]. The process may occur only rarely under natural conditions; the urinary bladder was fused to other organs in only 1.4% of the 1,254 cane toads that we collected from the field in tropical Australia. However, previous injuries or organ fusions incurred throughout a toad’s lifetime might not be detected at dissection if the healing process was complete. Physical damage may substantially increase the incidence of this mechanism; bladder abnormalities were observed in 17.5% of 40 toads that we subjected to intracoelomic needle penetration.

We found a variety of foreign materials in the coelom and urinary bladder of dissected wild cane toads. Foreign objects may enter the coelom via a variety of pathways: *(1)* oral route (e.g., an ingested wasp burrowed free of the stomach and partially occupied the coelom; several trophically transmitted parasite species are ingested and migrate to the exterior walls of the gut), *(2)* cloacal penetration (e.g., grass, leaves and twigs may have entered the bladder directly through the cloaca), *(3)* skin penetration (e.g., sharp objects such as sticks may have pierced the toad’s skin during hopping; several parasite species burrow directly through the skin and enter the coelom). Our findings suggest that regardless of their route of ingress, all of these foreign objects may be subsequently eliminated via the toad’s urinary bladder. Researchers planning to implant tracking and identification devices (e.g., radiotelemetry transmitters, passive integrated transponder [PIT] tags) intracoelomically should consider the likelihood that these devices will be shed into the environment via the urinary bladder. Shed PIT tags have been reported in some amphibians, but not others. For example, five of six wild gravid female *Ambystoma maculatum* rapidly (within five days of implantation) passed their PIT tags during egg-laying [14] and 56% of *Anaxyrus boreas boreas* passed their PIT tags within 9 weeks in the laboratory [15]. Conversely, five of five PIT tags implanted into captive *Rana temporaria* lasted longer than 19 months [16]. Viable alternatives to coelomic implantation of tracking and identification devices include affixing transmitters to the toad’s waist using customized belts (see [17]) and implanting PIT tags above the musculature in the toad’s back leg (see [18]), thereby avoiding contact with the urinary bladder.

We examined a subsample of the observed bladder abnormalities using histology. Acanthocephalan parasites were identified in the bladder walls of three toads. Acanthocephalans require two or more hosts to complete their life cycle [19]. Arthropods (mainly crustaceans, insects and myriapods) act as intermediate hosts, in which the egg hatches to release an acanthor, which develops into an acanthella. With the proboscis everted, this acanthella penetrates the gut wall of the invertebrate and moves into the body cavity, where it encysts to form the infective cystacanth. The cystacanth formed within the hyaline cyst wall is the final larval stage within the arthropod intermediate host, the hyaline covering apparently protecting it from host defenses. If this cystacanth is ingested by a paratenic host, it encysts again; if it is ingested by a suitable final host, it excysts, everts its proboscis, and pierces the gut wall, to feed and develop sexual organs. Adult acanthocephalans occur in all vertebrate classes and inhabit the intestines of their host [19]. The presence of a cystacanth in the urinary bladder of one cane toad suggests that its passage to the bladder was passive, and occurred before the proboscis was everted and further development occurred. The most plausible explanation for this observation is that, being in an abnormal host, this cyst was extruded from the intestine before it excysted, in a process similar to that described for inanimate objects by Tracy et al. [1], and transported to the urinary bladder. In several affected toads, fine strands of connective tissue (‘strings’) were noted running between the urinary bladder and other organs, in some cases connecting directly to, or adjacent to, parasitic cysts. However, no parasites were found inside these structures. We would have expected that, if the cane toads were abnormal hosts, the ingested larvae would either have been destroyed within the intestinal tract, or excreted in the feces. We could not confirm that this developmental stage would be excreted to the exterior via the bladder, as it is unlikely that any Acanthocephala within the lumen of the urinary bladder would be recovered in serial sections. Two bladder sections contained Acanthocephala in the process of being actively destroyed by the host defenses. The disposition of the spines indicated that the probosces were everted. No cyst wall was evident in those surrounded by dense inflammatory tissues. We were unable to determine whether the antecedent cystacanths excysted in the toads’ guts (as would occur in normal development) and were then transported to the bladders, or whether they were transported there in the cystic form (which then excysted and became exposed to the host’s defenses). The active destruction of non-encysted stages within the bladder wall indicates that the bladder plays an important, and hitherto unappreciated, role in control of this metazoan parasite. Finding these larval forms in the bladder wall of three toads suggests that this is a regular mechanism for isolating and excreting these parasites. In a series of operations Goodchild [20] demonstrated the plasticity of the amphibian gut in re-establishing function after ligation or resection of intestine. Spontaneous movements of the intestinal sections occur until the cut ends meet other organs, including the bladder and liver, and adhesions occur. We deduce that the passage of foreign objects, including Acanthocephala, is made possible by the plasticity and reconstructional ability of internal organs of these amphibians.

The species of Acanthocephala involved are not known. Acanthocephalans infect many taxa of Australian frogs, primarily ground-dwelling Myobatrachids [21]. No Acanthocephala were recovered from the intestines of 20 Green Tree Frogs (*Litoria caerulea*) collected from sites in tropical northern Australia, although their bladders were not examined [22]. A review of Australian Acanthocephala included cane toads as a paratenic host of *Porrorchis hylae* but the site of infection was not reported [23]. Dissections of *R*. *marina* from northern Australia (between 1989 and 1992) revealed only 7/794 possessed larval Acanthocephala in their intestinal tract, and in tropical Cape York Peninsula 7/52 contained unidentified adult Acanthocephala in their intestinal tract (D. Barton, pers. comm.). Encysted acanthocephalan larvae occurred in 10/166 cane toads dissected in Queensland, Australia [24]. Elsewhere in the cane toad’s introduced range, adult acanthocephalans (*Acanthocephalus bufonis*) infected the intestinal tract of 47/48 toads in Hawaii [25], larval *Pseudoacanthocephalu*s sp. were present in the small intestine of toads in Grenada [26] and a cystacanth occurred in the intestinal serosa of toads in Grenada [26]. Within its native range in Central and South America the intestines of *R*. *marina* are parasitized by two species of adult Acanthocephala: *Pseudoacanthocephalus lutzi* [27] and *Acanthocephalus correalimai* [28]. Cystacanths of the genus *Centrorhynchus* sp. were discovered encysted in the peritoneum of 11/49 cane toads in Mexico [29] and 2/40 cane toads had larval *Oncicola* sp. encysted in their mesentery in Mexico [30]. To our knowledge, the present study is the first to report any developmental stage of acanthocephalan occurring in the urinary bladder tissue of anurans [21]. Because bladder tissues are not routinely examined when dissecting hosts for parasites, metazoan parasites in these tissues could easily be overlooked.

In the present study, histological examination of bladder abnormalities revealed a variety of anomalies in some animals, yet in others the tissue appeared normal. The sections showing various pathology but no foreign matter may have been due to *(1)* foreign matter such as helminths having been already resorbed, *(2)* the sections not being sufficiently extensive to reveal foreign material, *(3)* or to other unrelated causes. We were unable to determine whether the range of pathological conditions in the toad bladders in which no parasites were found in serial sections, were in fact due to acanthocephalans, other metazoan parasites, or non-parasitic causes. No other metazoan parasites were seen in serial sections. Further studies concentrating on the urinary bladder may reveal that this organ is involved in the destruction or excretion of other helminth groups, as well as in a diverse suite of other foreign objects.

## Supporting Information

S1 Table Gross and histological appearance of urinary bladder abnormalities in cane toads. (PDF)
