# Supplementary material for: Wild Cane Toads (Rhinella marina) Expel Foreign Matter from the Coelom via the Urinary Bladder in Response to Internal Injury, Endoparasites and Disease
Source: PLoS One. 2015 Aug 12;10(8):e0134036. doi: 10.1371/journal.pone.0134036 (PMC4533966; doi:10.1371/journal.pone.0134036)
Supplement: S1 Table — (PDF) [file pone.0134036.s001.pdf]

Wild cane toads (*Rhinella marina*) expel foreign matter from the coelom via the urinary bladder in response to internal injury, endoparasites and disease.

Crystal Kelehear, Hugh I. Jones, Benjamin A. Wood, and Richard Shine.

S1 Table. Gross and histological appearance of urinary bladder abnormalities in cane toads.

Toad #	Appearance of abnormality
E103	Large grass seeds pierce right lung & are engulfed by urinary bladder (some are free inside the lumen), right lung is deflated & has lost elasticity
A1*	Part of liver & right lung is fused to urinary bladder via a thin string, a pentastomid (*Raillietiella frenata*) adjacent the fusion (inside the right lung) is dead & degrading; bladder wall has small cysts. Histology: nodule with fragments of everted acanthocephalan spines surrounded by dense concentric layers of inflammatory cells within urinary bladder wall
A12*	Fat body is fused to the mesentery, urinary bladder is fused to the fat body & mesentery, small cyst is present on mesentery at the point of fusion. Histology: urinary bladder is fused to mesentery; otherwise normal
BH55	Urinary bladder fused to growth on stomach wall
LT43	Urinary bladder fused to growth on stomach wall
W41	Urinary bladder fused to stomach wall
BH146	Urinary bladder fused to stomach wall
NA1	Urinary bladder fused to liver, liver is slimy, lumpy & atypically dark with brown & black blotches
W24	Urinary bladder fused to fat body, liver, & abdominal wall
ML230	Urinary bladder fused to liver & one lung
D30	Urinary bladder fused to mesentery adjacent pancreas
D236*	Urinary bladder fused to mesentery where there are several small cysts. Histology: normal, no cysts visible in sections
S13*	Urinary bladder fused to abdominal wall & liver, large mass free inside the bladder. Histology: prominent hemosiderin deposition with numerous macrophages in the liver

D233* Autonomous piece of fat is fused to the urinary bladder. Histology: normal.

D13 Urinary bladder is fused to a hard bubbly orange mass on the mesentery; large cyst is attached to mass

4041 Bladder fused to ovary & other organs; large lumps in mesentery adjacent to kidney, kidney inflamed & lumpy

ML76 Both ovaries are fused together to form one, urinary bladder adhered to ovaries; malodorous white mass inside bladder lumen

LT124 Both ovaries are fused together to form one, focal point of ovary fusion is joined to urinary bladder, at this adhesion point there is a clump of calcified leaves & sticks inside the bladder lumen

BH210 Soft mass free in lumen of urinary bladder

D215 Clumps of oily yellow material (likely fat) free in lumen of urinary bladder

A2* Cysts on urinary bladder wall. Histology: nodule containing acanthocephalan cystacanth with inverted spines, within hyaline capsule (Fig. 2a); moderate lymphohistiocytic inflammation at anterior pole; everted acanthocephalan spines embedded in mass of inflammatory cells (Fig. 2b); two cysts with concentric layers of inflammatory cells & fibroblasts surrounding resorbing trunks of larval acanthocephala

A4* Cyst on urinary bladder wall. Histology: normal; no cyst visible in sections

C1* Urinary bladder is fused to gall bladder & liver. Histology: hemosiderin & numerous macrophages

C2* Urinary bladder wall has two tiny cysts. Histology: lymphoid aggregates in urinary bladder wall; otherwise normal

C3* Autonomous piece of fat is fused to the urinary bladder. Histology: normal

C6* Urinary bladder is fused to small section of atypically dark liver. Histology: iron deposits in liver; foreign body (likely plant fragment) surrounded by inflammatory cells in urinary bladder wall

C8* Urinary bladder fused to liver adjacent to two lesions, bladder has dark intrusion (possibly liver tissue), liver is lumpy & discolored (Fig. 1a). Histology: iron deposits in liver

B4* Urinary bladder fused to liver via thin string

B5* Urinary bladder is fused to right abdominal wall, right fat body, & right lung; bladder wall has two granular growths. Histology: lymphoid aggregates in bladder wall; otherwise normal.

B7* Urinary bladder is fused to the mesentery via a thin string; several small cysts are on the adjacent mesentery (Fig. 1b); Histology: the thin string contains focal lymphoid aggregations; there is a hole in the urinary bladder tissue (possibly from a dislodged parasite)

B8* Urinary bladder is fused to upper abdominal wall by a thin string, a separate string connects urinary bladder to liver & right lung. Histology: area of fusion showed fat necrosis & xanthogranulomatous cystitis with hemosiderin deposition & lymphoid aggregates in the bladder wall; one of the thin strings contained hemosiderin-laden macrophages

B12* Urinary bladder wall has a small cyst. Histology: oblique longitudinal section of acanthocephalan trunk, in the process of being resorbed, surrounded by concentric layers of inflammatory cells

B17* Autonomous piece of red (vascularized) fat is fused to the urinary bladder. Histology: normal

B18* Urinary bladder wall has a small cyst. Histology: thick-walled cyst surrounds almost entirely resorbed trunk of acanthocephalan

B24 Autonomous piece of fat is fused to the urinary bladder

B34* Urinary bladder is adhered to spirurid cyst on stomach wall (Fig. 1c). Histology: spirurid cyst not present in sections examined; carcinoid-like cyst on stomach wall; otherwise normal

T2 Bladder fused to left Bidder's organ, both testes extremely segmented

T3 Urinary bladder fused to liver

T8 Urinary bladder fused to liver by a thin string

* Denotes tissue that was processed for histology
